# Lions & sea lions & bears, oh my: utilizing museum specimens to study the ossification sequence of carnivoran taxa

**DOI:** 10.1186/s40850-024-00201-3

**Published:** 2024-04-29

**Authors:** Jonathan L. Sarasa, Alexander S. Okamoto, Mark A. Wright, Stephanie E. Pierce, Terence D. Capellini

**Affiliations:** 1 University of Michigan, Ann Arbor, MI USA; 2 Museum of Comparative Zoology, Department of Organismic and Evolutionary Biology, Harvard University, Cambridge, MA USA; 3 Human Evolutionary Biology, Harvard University, Cambridge, MA USA; 4 Broad Institute of Harvard and MIT, Cambridge, MA USA

**Keywords:** Ossification, Skeleton, Carnivora, Micro-computed tomography, Prenatal, Museum specimens, Feliformia, Caniformia

## Abstract

**Background:**

Mammalian skeletons are largely formed before birth. Heterochronic changes in skeletal formation can be investigated by comparing the order of ossification for different elements of the skeleton. Due to the challenge of collecting prenatal specimens in viviparous taxa, opportunistically collected museum specimens provide the best material for studying prenatal skeletal development across many mammalian species. Previous studies have investigated ossification sequence in a range of mammalian species, but little is known about the pattern of bone formation in Carnivora. Carnivorans have diverse ecologies, diets, and biomechanical specializations and are well-suited for investigating questions in evolutionary biology. Currently, developmental data on carnivorans is largely limited to domesticated species. To expand available data on carnivoran skeletal development, we used micro-computed tomography (micro-CT) to non-invasively evaluate the degree of ossification in all prenatal carnivoran specimens housed in the Harvard Museum of Comparative Zoology. By coding the presence or absence of bones in each specimen, we constructed ossification sequences for each species. Parsimov-based genetic inference (PGi) was then used to identify heterochronic shifts between carnivoran lineages and reconstruct the ancestral ossification sequence of Carnivora.

**Results:**

We used micro-CT to study prenatal ossification sequence in six carnivora species: *Eumetopias jubatus* (Steller sea lion, *n* = 6), *Herpestes javanicus* (small Indian mongoose, *n* = 1), *Panthera leo* (lion, *n* = 1), *Urocyon cinereoargenteus* (gray fox, *n* = 1), *Ursus arctos arctos* (Eurasian brown bear, *n* = 1), and *Viverricula indica* (small Indian civet, *n* = 5). Due to the relatively later stage of collection for the available specimens, few heterochronic shifts were identified. Ossification sequences of feliform species showed complete agreement with the domestic cat. In caniforms, the bear and fox ossification sequences largely matched the dog, but numerous heterochronic shifts were identified in the sea lion.

**Conclusions:**

We use museum specimens to generate cranial and postcranial micro-CT data on six species split between the two major carnivoran clades: Caniformia and Feliformia. Our data suggest that the ossification sequence of domestic dogs and cats are likely good models for terrestrial caniforms and feliforms, respectively, but not pinnipeds.

**Supplementary Information:**

The online version contains supplementary material available at 10.1186/s40850-024-00201-3.

## Background

Prenatal specimens of most mammalian species are difficult to acquire due to the ethical and technical challenges of collecting embryos in viviparous animals. One solution is the establishment of breeding colonies [1, 2], however, this is unfeasible for most species given their larger sizes, long gestational cycles, and/or endangered status, among other factors. Alternatively, natural history museums house opportunistically collected prenatal specimens obtained from wild-caught and zoo-housed animals [3–6]. Most species in a given collection will be represented by only a few–if any–specimens, and these will generally be later stages when pregnancy of the mother would have been clearly observable. These stages often correspond with the developmental window where the skeleton begins to ossify, either via direct intramembranous bone formation or a cartilage template [7]. The high density of bone means that modern methods such as micro-computed tomography (micro-CT) can be used to visualize the developing skeleton without damaging the specimen.

Analysis of the sequence of developmental events is a powerful method for detecting heterochronic changes between species using specimens of unknown age [8]. Previous studies have often focused on changes in skeletal structure at a broad phylogenetic scale to reconstruct ossification patterns across the mammalian tree [9–11]. Studies examining heterochronies in specific clades have been largely focused on marsupials [5, 12–14] or bats [15–17], identifying accelerated ossification in the skull and forelimbs to facilitate precocial birth in the former, and prolonged fore- and hind limb development as adaptations for flight in the latter. For most eutherian clades, however, ossification sequence data is limited to only a few representative species.

Carnivora is one clade that has previously received little attention in comparative ossification studies. Contrary to its name, the clade Carnivora contains species with a wide-range of diets and ecologies [18, 19]. Accordingly, Carnivora is morphologically diverse and has a large geographic distribution, with species native to five continents and all oceans [18]. Many carnivoran species, particularly those with predatory lifestyles, face an elevated extinction risk compared to other mammals [20], and, largely due to charismatic megafauna, carnivorans are often a focus of education and conservation. This clade also offers an attractive system for studying adaptations in morphology, locomotion, and behavior [18]. While there are almost 300 extant species of Carnivora, detailed ossification sequence information is available for only two species, the domestic dog [21] and cat [22, 23] both of which have been subjected to artificial selection over the past thousands of years [24]. Information on other non-domesticated species includes prenatal skull data based on a few specimens of *Mustela sp., Phoca sp.*, and *Eumetopias jubatus* [15], as well as full skeletal scans of caniform neonatal/late-stage specimens with a focus on ursids [25]. While domestic dogs and cats represent the two main clades within Carnivora, the dog-like Caniformia (e.g., bears, seals, wolves) and the cat-like Feliformia (e.g., big cats, civets, hyenas) respectively, which diverged around 40 million years ago [26], it is unlikely that their ossification sequences are representative of all species in their respective lineages.

**Fig. 1:**
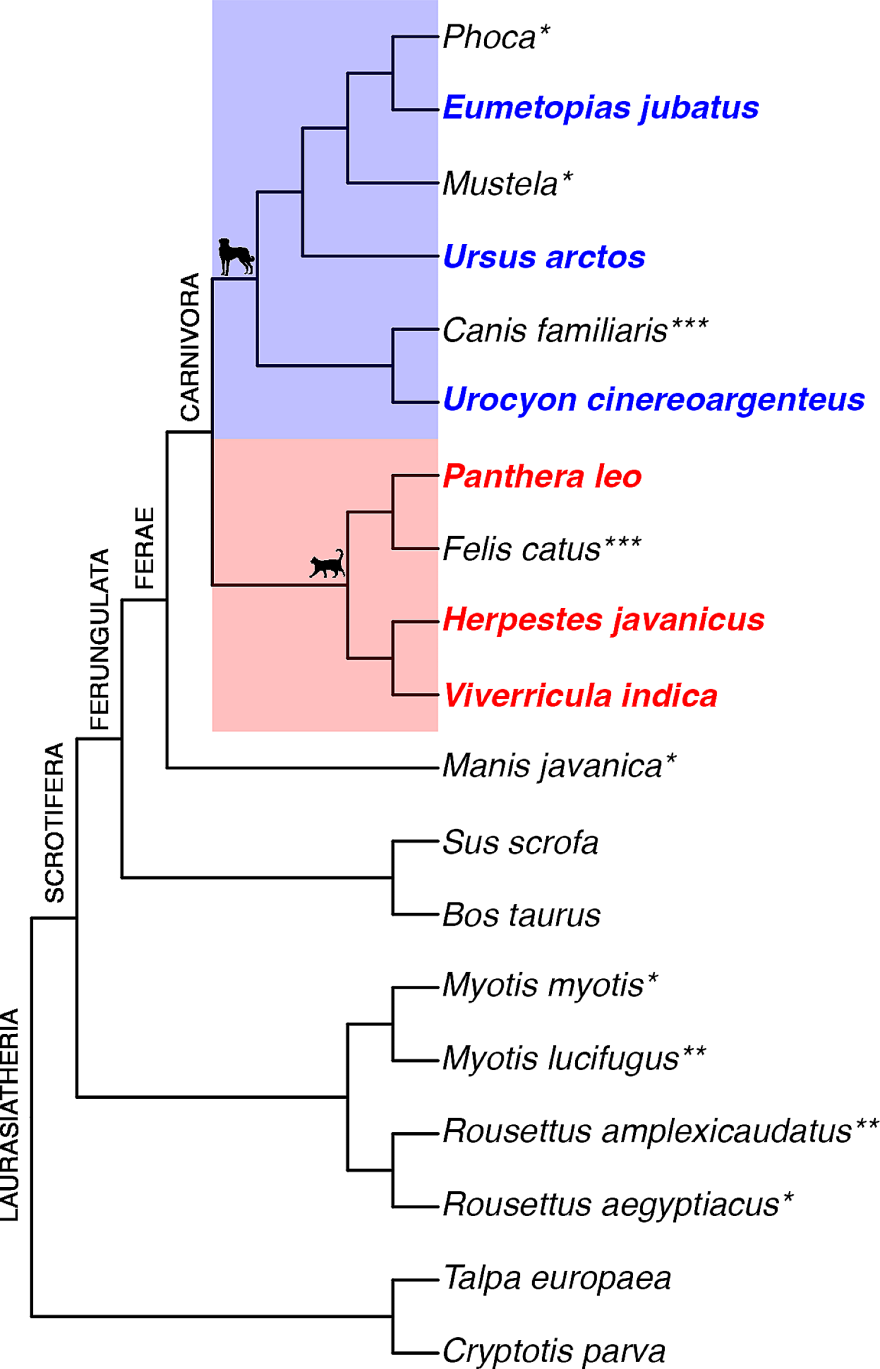
Cladogram of carnivoran species with available ossification sequence data. Carnivora is divided into two major clades, Caniformia (blue box, dog silhouette) and Feliformia (red box, cat silhouette). New caniform species included in this study are highlighted in bold blue text and new feliforms are in bold red text. *indicates only cranial data is available for this species. **indicates only postcranial data is available. ***indicates domesticated species. Silhouettes are from phylopic.org

**Fig. 2:**
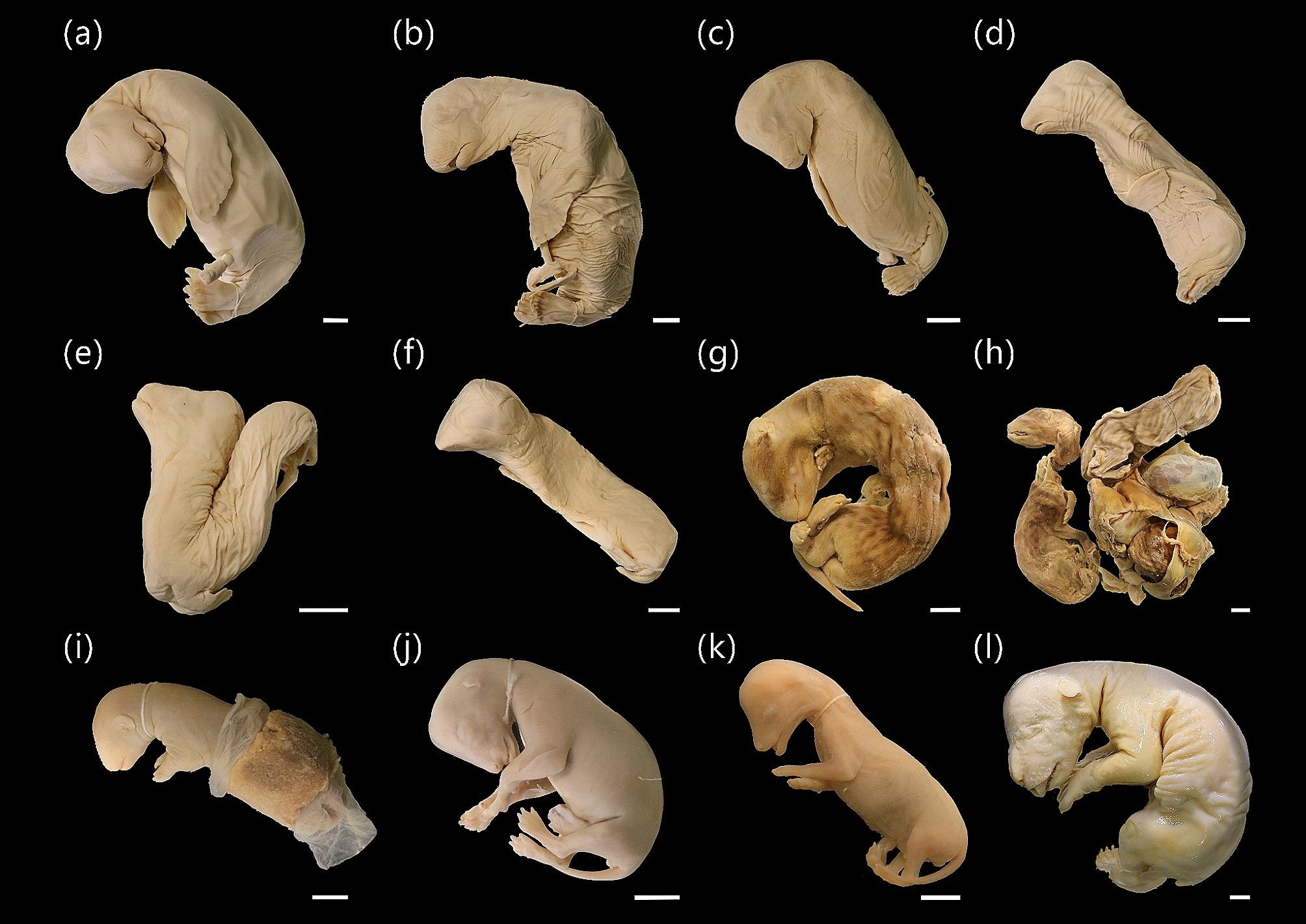
Lateral view of *Eumetopias jubatus* specimens, MCZ: Mamm:56920, 56934–56937, 56776 (**a**-**f**). Lateral view of other Carnivora scanned for this study: *Viverricula indica*, MCZ: Mamm:45565, 45786 (**g**, **h**), *Herpestes javanicus*, MCZ: Mamm:64635 (**i**), *Panthera leo*, MCZ: Mamm:56777 (**j**), *Urocyon cinereoargenteus*, MCZ: Mamm:64708 (**k**), and *Ursus arctos arctos*, MCZ: Mamm:14958 (**l**). Specimens were not removed from any remaining uterine tissues, (**h**, **i**, **g**, **k**, & **l**) have been flipped horizontally. White scale bars = 10 mm

**Fig. 3:**
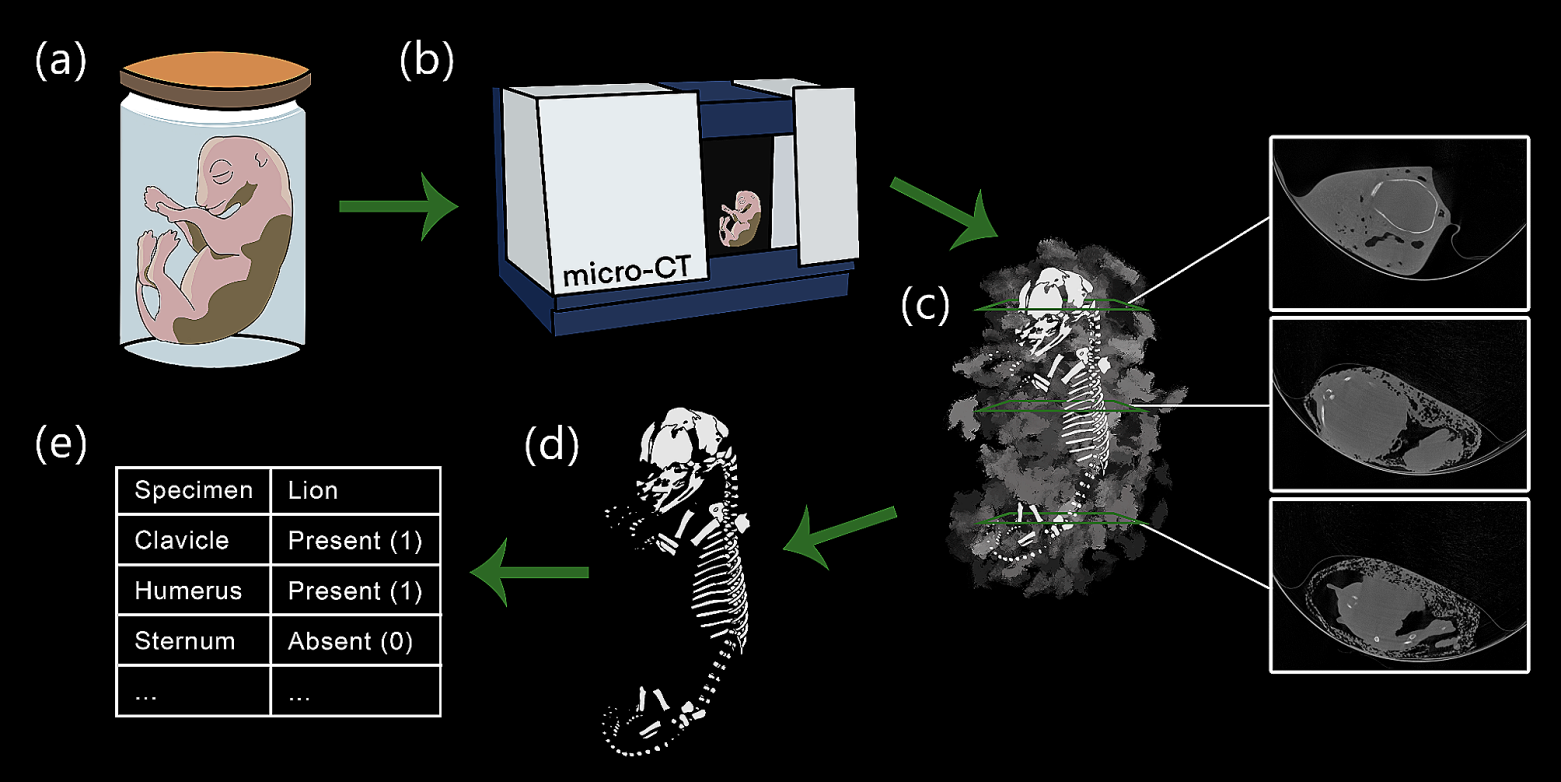
Process for collecting ossification data on museum specimens. The lion embryo (MCZ: Mamm:56777) is used as an example. This specimen has been stored in 100% ethanol in the MCZ wet collections (**a**). The specimen undergoes micro-CT scanning in the Bruker Skyscan 1273 (**b**). The reconstructed output of the CT-scan consists of 2D slices containing background noise (**c**) and requires segmentation to create a 3D model of the developing skeleton (**d**). The presence or absence of each bone is scored based on the 3D model (**e**)

Our study aims to extend available data on prenatal skeletal development in carnivorans. To this end, we micro-CT scanned all prenatal carnivoran specimens housed in the Harvard Museum of Comparative Zoology (MZC) (Figs. 1 and 2, Table S1). After segmentation of each scan, we noted the presence or absence of bones to create a cranial and postcranial ossification sequence for each species (Fig. 3). Then, we ran Parsimov-based genetic inference (PGi) analysis (Methods) on the cranial and postcranial datasets to identify ancestral ossification sequences and heterochronic changes within Carnivora.

## Results

### Ossification status of analyzed specimens

**Fig. 4:**
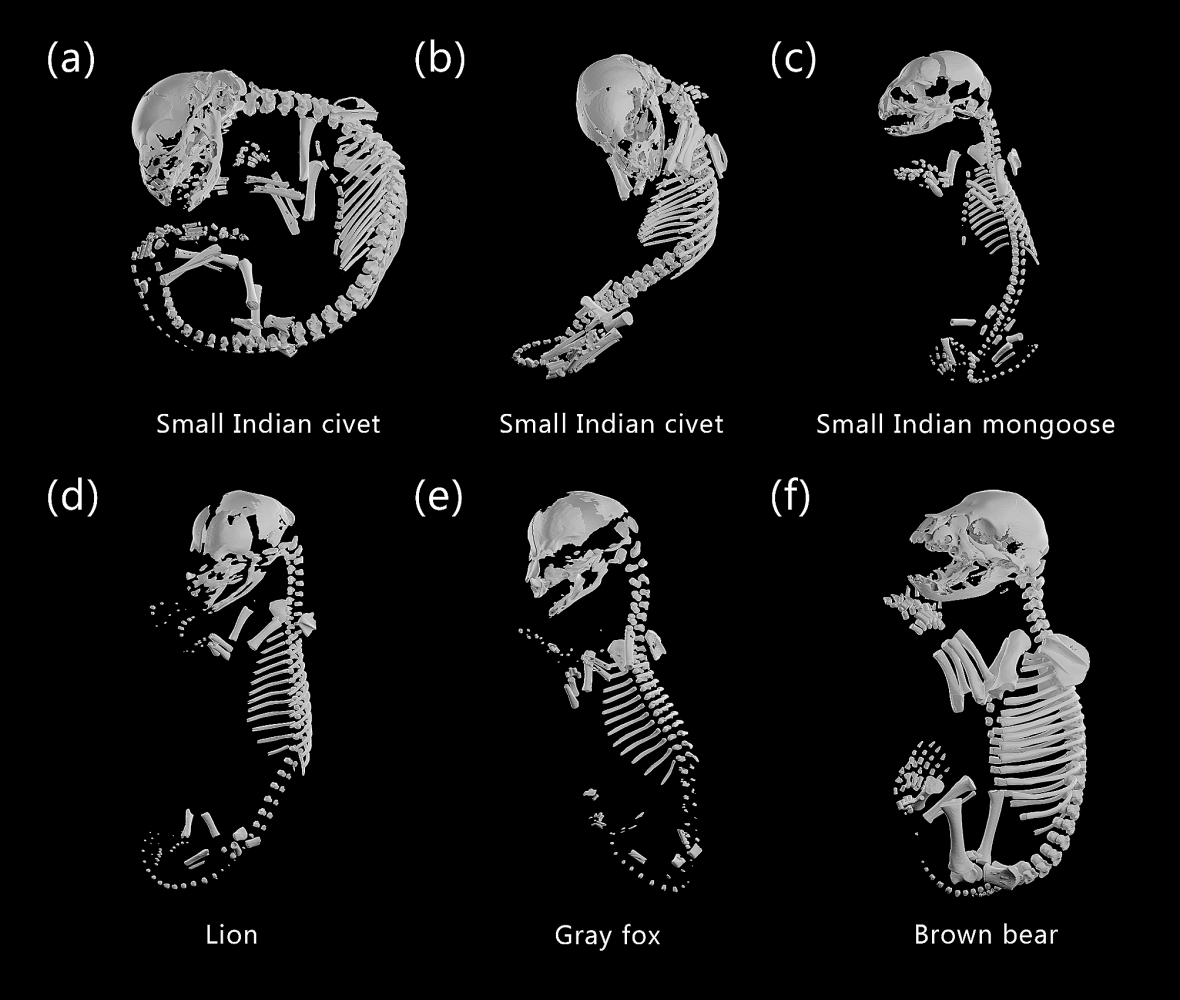
Skeletal reconstructions of prenatal carnivoran species using micro-CT. Skeletons include *Viverricula indica* (small Indian civet), MCZ: Mamm:45565, 45786 (**a**, **b**), *Herpestes javanicus* (small Indian mongoose), MCZ: Mamm:64635 (**c**), *Panthera leo* (lion), MCZ: Mamm:56777 (**d**), *Urocyon cinereoargenteus* (gray fox), MCZ: Mamm:64708 (**e**), and *Ursus arctos arctos* (Eurasian brown bear), MCZ: Mamm:14958 (**f**). Species are shown in lateral view to the extent possible given the position of the specimens. Images not to scale. The texture used in the reconstruction renders was made by Katsukagi on 3dtextures.me

**Fig. 5:**
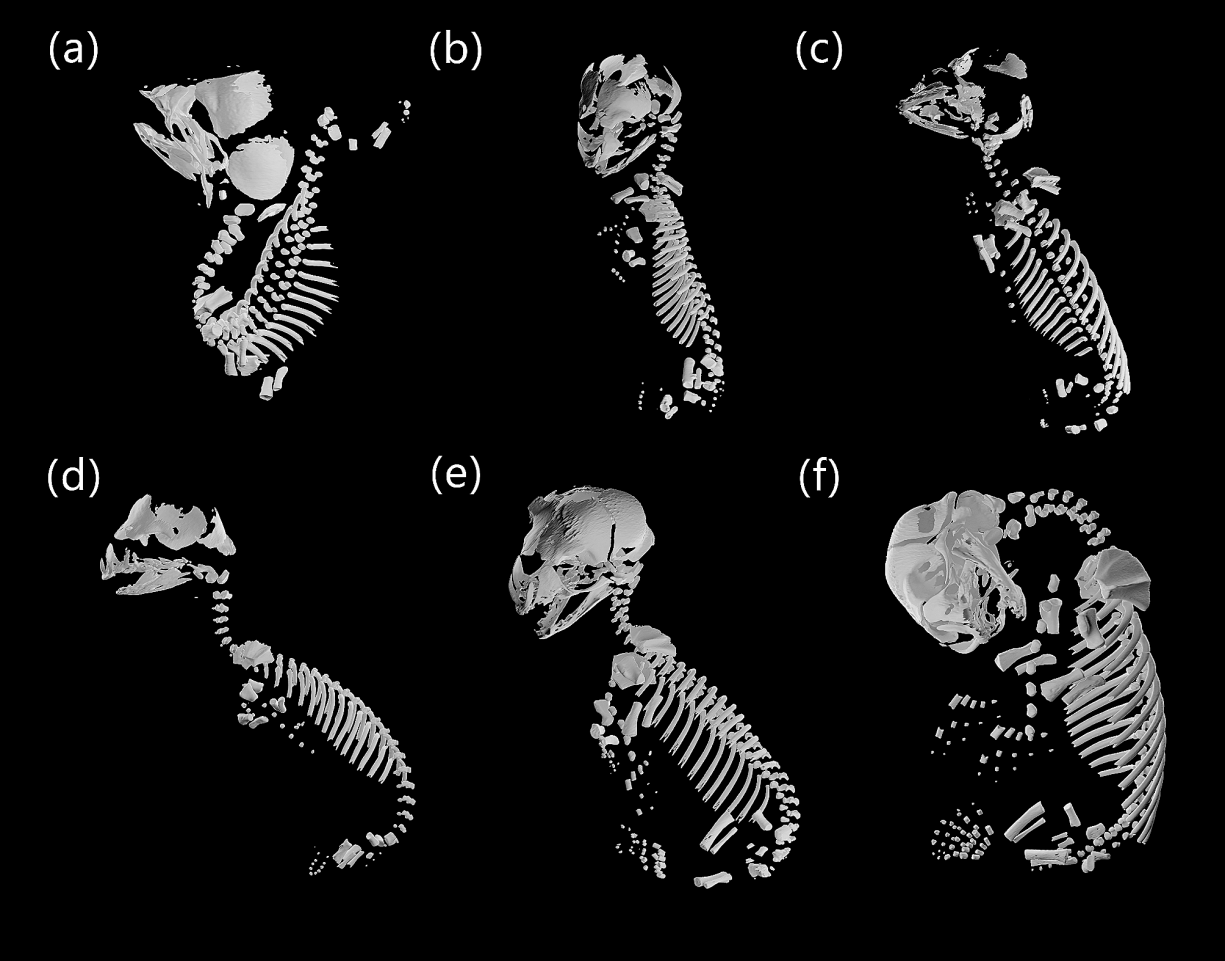
Skeletal reconstructions of prenatal *Eumetopias jubatus* (Steller sea lion) using micro-CT. These specimens span three developmental stages, with MCZ: Mamm:56936 showing the most immature skeleton (**a**), MCZ: Mamm:56934, 56937, 56935 showing an intermediate stage (**b**, **c**, **d**), and MCZ: Mamm:56920, 56776 (**e**, **f**) showing the highest level of ossification. Species are shown in lateral view to the extent possible given the position of the specimens. **f** has been flipped horizontally. Images not to scale. The texture used in the reconstruction renders was made by Katsukagi on 3dtextures.me

We micro-CT scanned fifteen carnivoran specimens of six species: *Eumetopias jubatus* (Steller sea lion, *n* = 6), *Herpestes javanicus* (small Indian mongoose, *n* = 1), *Panthera leo* (lion, *n* = 1), *Urocyon cinereoargenteus* (gray fox, *n* = 1), *Ursus arctos arctos* (Eurasian brown bear, *n* = 1), and *Viverricula indica* (small Indian civet, *n* = 5) (Figs. 4 and 5). All specimens had already undergone significant amounts of ossification, with at least 70% of the skeleton present (Table S2). The *V. indica* and *U. arctos arctos* specimens were completely ossified except for the carpals (Fig. 4a, b,f). As there was no ossification variation in the four sibling *V. indica* specimens (all MCZ: Mamm:45786), only a single representative specimen was fully segmented. The *U. arctos arctos* specimen cannot be clearly distinguished from neonatal ursid specimens [25], suggesting it was collected close to birth. The six specimens of *E. jubatus* could be clearly divided into three developmental stages (Fig. 5); MCZ: Mamm:56936 was the least developed, lacking ossification of the phalanges, metapodials, sternum, caudal vertebrae, pubis, and ischium (Fig. 5a). MCZ: Mamm:56934, MCZ: Mamm:56935, and MCZ: Mamm:56937 were intermediate, with mixed ossification patterns in the manual and pedal phalanges as well as the pubis, which could not be resolved into a single consistent sequence (Fig. 5b-d). MCZ: Mamm:56920 and MCZ: Mamm:56776 were the oldest, with larger total body size, clear ossification of the bones defining the intermediate stage, presence of the nasal bone, and well-developed phalanges (Figs. 2 and 5e-f).

### Ossification sequence heterochrony and ancestral state reconstruction

**Fig. 6:**
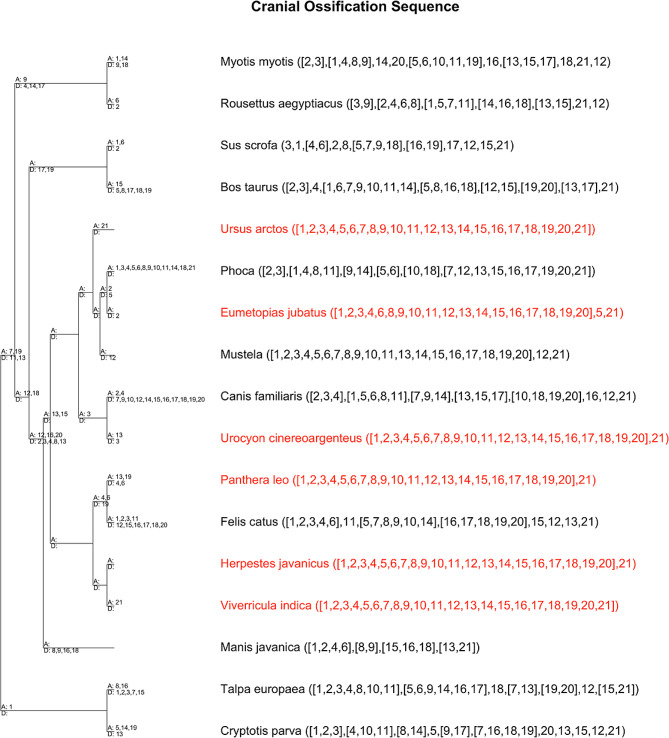
PGi consensus tree of cranial characters. Ossification sequence is shown for each tip. Species with new data shown in red. Character key: 1: Premaxilla, 2: Maxilla, 3: Dentary, 4: Frontal, 5: Nasal, 6: Jugal, 7: Lacrimal, 8: Parietal, 9: Squamosal, 10: Vomer, 11: Palatine, 12: Orbitosphenoid, 13: Basisphenoid, 14: Pterygoid, 15: Alisphenoid, 16: Basioccipital, 17: Supraoccipital, 18: Exoccipital, 19: Ectotympanic, 20: Goniale, 21: Petrosal. A = advanced ossification, D = delayed ossification

**Fig. 7:**
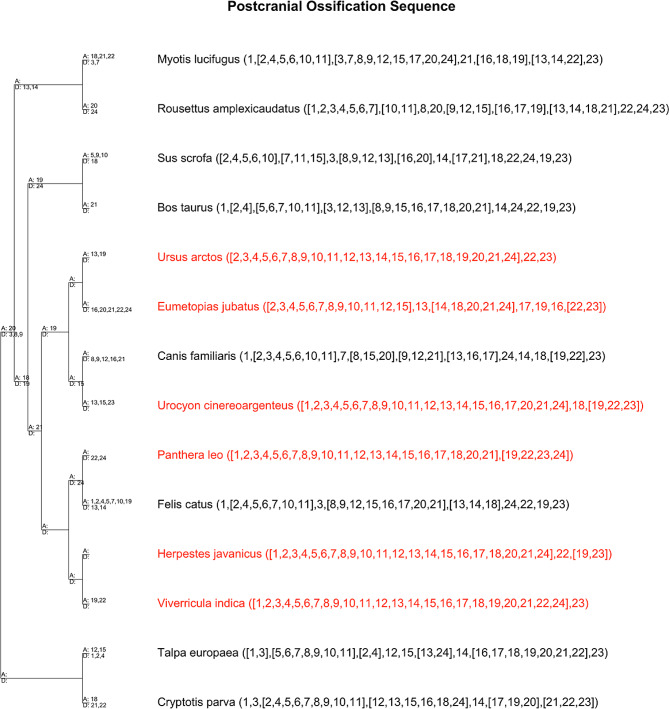
PGi consensus tree of postcranial characters. Ossification sequence is shown for each tip. Species with new data shown in red. Character key: 1: Clavicle, 2: Humerus, 3: Ribs, 4: Femur, 5: Radius, 6: Ulna, 7: Scapula, 8: Cervical vertebrae, 9: Thoracic vertebrae, 10: Tibia, 11: Fibula, 12: Lumbar vertebrae, 13: Sacral vertebrae, 14: Caudal vertebrae, 15: Ilium, 16: Manual phalanges, 17: Pedal phalanges, 18: Ischium, 19: Pubis, 20: Metacarpals, 21: Metatarsals, 22: Tarsals, 23: Carpals, 24: Sternum. A = advanced ossification, D = delayed ossification

Parsimov-based genetic inference (PGi) analysis of the cranial data resulted in eight trees of similar raw tree lengths: 159, 163, 165, 170, 161, 166, 165, and 160. PGi analysis of the postcranial data also produced eight trees of similar raw lengths: 120, 118, 119, 118, 121, 121, 117, and 122, indicating that the parameters chosen were appropriate for both matrices. The consensus of the shortest tree(s) for each analysis was used for identifying heterochronic shifts in ossification sequence (Figs. 6 and 7). Since the skull was largely developed in all new taxa in our dataset, the ancestral carnivoran cranial ossification sequence could only be resolved into two stages, with the alisphenoid and petrosal forming after all other elements. This same sequence is reconstructed for the ancestral caniform and feliform, and while this is consistent with cat ossification sequence, the alisphenoid forms well before the petrosal in dogs, suggesting that the finding of simultaneity is likely artifactual. The only clear heterochrony identified in the skull is that specimens of *E. jubatus* show delayed formation of the nasal bone compared to *Phoca sp.* as well as domestic dog and cat.

The reconstructed ancestral carnivoran postcranial ossification sequence was resolved to seven stages: 1: clavicle, 2: humerus, ribs, femur, radius, ulna, scapula, cervical vertebrae, thoracic vertebrae, tibia, fibula, lumbar vertebrae, ilium, manual phalanges, metacarpals, metatarsals, sternum, 3: sacral vertebrae, caudal vertebrae, 4: pedal phalanges, 5: ischium, 6: tarsals, 7: pubis, carpals. The caniform and feliform ancestors had less sequence resolution, with six and four distinct stages, respectively. The caniform ancestral sequence was: 1: clavicle, 2: humerus, ribs, femur, radius, ulna, scapula, cervical vertebrae, thoracic vertebrae, tibia, fibula, lumbar vertebrae, ilium, manual phalanges, metacarpals, metatarsals, sternum, 3: sacral vertebrae, caudal vertebrae, ischium, 4: pedal phalanges, 5: tarsals, pubis, 6: carpals. The feliform ancestral sequence was: 1: clavicle, humerus, ribs, femur, radius, ulna, scapula, cervical vertebrae, thoracic vertebrae, tibia, fibula, lumbar vertebrae, sacral vertebrae, caudal vertebrae, ilium, manual phalanges, pedal phalanges, metacarpals, metatarsals, sternum, 2: ischium, 3: tarsals, 4; pubis, carpals. All feliform species included in this study were completely compatible with the ossification sequence reported for the domestic cat in the skull and postcranial skeleton. Within Caniformia, *U. cinereoargenteus* was similarly compatible with dog. In *U. arctos arctos*, the pubis is well formed while only a single small tarsal has begun to ossify in each foot, suggesting that in the bear the pubis forms before the tarsals in contrast to dog where these bones are reported to appear simultaneously. This could be either a true heterochrony or a lack of sequence resolution for later stages in dog. The ossification sequence of *E. jubatus* has small differences with the ossification sequence of dog (and cat) in both the skull and postcranial skeleton. Heterochronic shifts identified for *E. jubatus* compared to the caniform ancestor include delays in the ossification of the manual phalanges, metacarpals, metatarsals, tarsals, and sternum, with the manual phalanges and metatarsals showing the clearest delays relative to dog when the ossification sequences are compared directly.

## Discussion

Even a single prenatal specimen can give insights into the ossification sequence of a species through observation of the presence or absence of bones seen in the adult skeleton. In this way, data can be generated for species with limited prenatal material which can then be evaluated in light of related species for which more complete datasets are available. The data presented in this study suggest that the ossification sequence of the domestic cat is largely shared by other feliform lineages, at least for the later stages of skeletal development [22, 23]. Within Caniformia, the gray fox specimen completely agrees with the domestic dog ossification sequence at later stages and the bear is also a close match [21]. In contrast, the Steller sea lion ossification sequence shows a number of differences to dog in both the skull and postcranial skeleton. The cranial sequence identified here is consistent with the data included in Koyabu et al. [15] on a single *E. jubatus* specimen but distinct from *Phoca.* We also note that there is variation within the relative ossification timing of the manual phalanges, pedal phalanges, and pubis in *E. jubatus.* This could be due to sex differences [27], which were not investigated in this study since information on the sex of the specimens was not available, or just natural variation in ossification sequence within the species. These findings are consistent with a study on cranial suture closures in carnivorans which found that pinnipeds had a very high rate of interspecific heterochrony and that Caniformia contains more cranial diversity than Feliformia [28]. It is perhaps unsurprising that the largest variation in ossification sequence in our analysis corresponds to an ecological transition from terrestrial to aquatic, given that the medium in which an animal moves plays an outsized role in influencing efficient structural design [29]. Similarly, domestic dogs are more variable than domestic cats [30], even showing slight differences in ossification between breeds in neonates [31] albeit this may not be unexpected considering the extensive breeding strategies used by breeders to optimize genetically heritable traits important for different functionalities. Regardless, the greater variability in dogs has been hypothesized to extend to Caniformia generally [28] and this agrees with our ossification sequence results.

Unfortunately, the MCZ collection only contains specimen(s) belonging to a single stage for a majority of the species included in this study, providing a limited, yet important window into skeletal development. This limits the conclusions that can be drawn from PGi analysis since taxa with largely unresolved sequences are weighted the same as species with more complete sampling [32]. This results in poorly resolved ancestral ossification sequences and numerous heterochronies identified on internal branches which are artificial (Figs. 6 and 7). To account for this limitation, results presented in the main text are interpreted in the context of species with highly resolved sequences (generally dog or cat). On the other hand, these data add confidence to findings related to the bones which ossify relatively late in all carnivorans, such as the pubis, carpals, tarsals, and petrosal. The late ossification of these bones is consistent with other placental mammals [11, 15].

The timing and pattern of ossification for a given species can help reveal the biological mechanisms underlying unique anatomical structures, such as the unusual number of cervical vertebrae in manatees [33] and sloths [34], the elongated hind limbs of the jerboa [35], or proximal femur patterning differences between mammals [36]. Museum specimens can even be used to understand the development of species such as the Tasmanian tiger which have recently gone extinct [4]. A major limitation to studies on prenatal skeletal development is the challenge of assembling a sufficient sample size for a given taxon, which are often dispersed across museums worldwide and cannot be easily identified or staged remotely. To facilitate the use of prenatal museum specimens in research, it is critical that these specimens be thoroughly cataloged, and, where possible, detailed pictures and CT-scans be made publicly available via online repositories such as MorphoSource, MorphoMuseuM, or Dryad [37–40]. While limited in resolution, the six species included in this study span multiple carnivoran lineages which have not been previously investigated in terms of ossification sequence. Previous studies regarding the ontology of these species have been limited to neonatal or older specimens [25, 41–45]. By making the carnivoran specimens in the MCZ publicly available, we hope that future studies of these species can be extended to include prenatal development.

## Conclusions

Museum collections often contain rare, intact mammalian embryos preserved in fluid which can provide novel insights into skeletal development through micro-CT. In this study, we scanned and provide prenatal cranial and postcranial ossification data for six carnivoran species. We show that the ossification sequence of domestic dogs and cats is likely a good model for terrestrial caniforms and feliforms, respectively, but not pinnipeds, at least during the later stages of skeletal ossification. Based on our sampling, we suggest that ecological habits – terrestrial versus aquatic – may influence prenatal development in Carnivora.

## Methods

### Specimen identification and scanning

All specimens used in this study are housed in the Harvard Museum of Comparative Zoology (MCZ) Mammalogy (Mamm) fluid collections (Fig. 2). The Integrated Taxonomic Information System database (www.itis.gov) was used to identify valid genus and species names for all samples. While information on the age of these specimens is unavailable, specimens were identified as prenatal via an MCZ database (https://mczbase.mcz.harvard.edu/*)* query for the terms “embryo”, “embryos,” “fetus,” “fetuses” or visual inspection of the collections. Micro-CT was used to non-invasively evaluate the developing skeleton of each specimen. All scans were performed using a Bruker Skyscan 1273 in the MCZ Digital Imaging Facility with a voltage of 70 kV and current of 300 µA. Amongst scans, the resolution varied between 38.9997 μm and 51.652 μm to accommodate specimen size (Table S1). Scans were reconstructed as image stacks (.tif) using NRecon (Micro Photonics).

After reconstruction, micro-CT-scans were segmented in Amira-Avizo. The segmentation threshold was chosen separately for each scan to clearly distinguish ossified material from other tissues and background noise. Each bone was manually inspected using both the original reconstruction and segmented mesh to confirm the definitive presence or absence of each bone. Two specimens – MCZ: Mamm:61863 (*Pekania pennanti*) and MCZ: Mamm:61882 (*Vulpes vulpes*) – showed no ossification and were not considered further.

### Ossification sequence data

To evaluate the new specimens in the context of larger datasets, we coded the 21 cranial elements used by Koyabu et al. [15] and 22 postcranial elements used in Hautier et al. [11]. See Spiekman & Werneburg [5] and Li & Smith [25] for detailed figures showing cranial anatomy in marsupial embryos and neonatal carnivores, respectively. In keeping with previous studies, these two character sets were analyzed independently [1, 3, 11]. For each specimen, we coded whether each element was absent (0), partial (0.5), or present (1) (Table S2). Based on the absence or presence of ossified bones known to be present in the adult, we constructed an ossification sequence for each species. Elements with an ambiguous order due to intraspecific variation were coded as simultaneous. The ossification sequences generated in this study were added to data from existing studies to construct cranial and postcranial matrices [11, 15, 21–23]. Outgroup species were included from as many major lineages within Laurasiatheria as possible but not all possible outgroup species within each lineage were included due to the limits of PGi for resolving large datasets [32]. Included outgroup species had highly resolved ossification sequences and available cranial and postcranial data. These species included two chiropteran genera, *Myotis* and *Rousettus* (*M. myotis* and *R. aegyptiacus* for cranial, *M. lucifugus* and *R. amplexicaudatus* for postcranial), two artiodactyls (*Sus scrofa* and *Bos taurus)*, two eulipotyphlae (*Talpa europaea* and *Cryptotis parva)*, and one pholidotid *(Manis javanica*, cranial only). Carnivoran species included those investigated in this study as well as dogs and cats for all elements and *Phoca sp.*, and *Mustela sp.* for cranial only (Fig. 1). This resulted in a cranial matrix of 21 characters for 17 taxa (Table S3) and postcranial matrix of 22 characters for 14 taxa (Table S4). These datasets were imported into the R statistical environment (version 4.3.1) [46] and all ossification sequences converted to dense ranks using the *dense_rank* function from the *dplyr* package (version 1.1.2) [47]. A phylogenetic tree for all species included in the dataset was retrieved from TimeTree [48] and visualized using the *ggtree* package (version 3.8.2) [49]. The character matrix and phylogenetic tree for the cranial and postcranial datasets were converted to nexus file format for PGi analysis using the functions *write.nexus.data* and *write.nexus*, respectively, from the *Ape* package (version 5.7-1) [50].

### Identification of heterochronic changes

Heterochrony and ancestral ossification sequence reconstruction for internal nodes were assessed using Parsimov-based genetic inference (PGi) [32]. PGi was implemented in R via the *PGi2* package (https://github.com/lukebharrison/PGi2*).* PGi uses a dynamic programming approach and treats event sequence as a single complex character. PGi used a simplified genetic algorithm-based heuristic with a Parsimov edit cost function. This approach is replicated numerous times and consensus-methods are used to identify sequence heterochronies along the phylogenetic tree. The cranial and postcranial matrices were each analyzed using PGi with nruns = 8, cycles = 200, replicates = 200, ret.anc.seq = 200 and the semi-exhaustive approach with semi.ex.con.max.*n* = 2000. The consensus tree of the shortest trees generated by each run was calculated using *pgi.supercon* and visualized with *plot.pgi.tree*.

### Electronic supplementary material

Below is the link to the electronic supplementary material.

Supplementary Material 1

## Data Availability

All micro-CT data supporting the conclusions of this article are available as reconstructed TIF stacks and meshes in the MorphoSource repository, (https://www.morphosource.org/projects/000542707). All data derived from these scans are available in this paper or in the supplementary materials.

## Abbreviations

MCZ: Harvard Museum of Comparative Zoology
Mamm: Mammalogy
micro-CT: micro-computed tomography
PGi: Parsimov-based genetic inference
